# Differential gene expression underpinning the production of distinct sperm morphs in the wax moth *Galleria mellonella*

**DOI:** 10.1098/rsob.240002

**Published:** 2024-07-31

**Authors:** Emma Moth, Fiona Messer, Saurabh Chaudhary, Helen White-Cooper

**Affiliations:** ^1^ School of Biosciences, Cardiff University, Museum Avenue, Cardiff CF10 3AT, UK; ^2^ School of Biosciences, University of Sheffield, Sheffield S10 2TN, UK

**Keywords:** spermatogenesis, Lepidoptera, gene expression, transcriptome

## Abstract

Male Lepidoptera produce two distinct sperm types; each ejaculate contains both eupyrene sperm, which can fertilize the egg, and apyrene sperm, which are not fertilization competent. These sperm have distinct morphologies, unique functions and different proteomes. Their production is highly regulated, however, very few genes with specific roles in the production of one or other morph have been described. We present the first comparative transcriptomics study of precursors of eupyrene and apyrene sperm to identify genes potentially implicated in regulating or enacting the distinct differentiation programmes. Differentially expressed genes included genes with potential roles in transcriptional regulation, cell cycle and sperm morphology. We identified gene duplications generating paralogues with functions restricted to one or other morph. However, phylogenetic analysis also revealed evolutionary flexibility in expression patterns of duplicated genes between different lepidopteran species. An improved understanding of lepidopteran reproduction will be vital in targeting prevalent pests in agriculture, and on the flip side, ensuring the fertility and thus survival of pollinator populations in response to environmental stress.

## 1. Introduction

Spermatogenesis is a highly regulated process that results in the generation of mature sperm with highly specialized morphology. In insects, the process typically involves continued production of sperm throughout the adult life of the male, sustained by a male germline stem cell population. Stem cell divisions generate spermatogonia committed to differentiation, which then undergo mitotic amplification before differentiating into primary spermatocytes and switching to a meiotic cell cycle. Post-meiotic morphological changes include spermatid elongation, nuclear reshaping, mitochondrial reorganization and growth of the axoneme. Each spermatogonium is enveloped by somatic cyst cells that form a squamous epithelium within which the germline cells differentiate. In Lepidoptera, each male makes two distinct sperm types, a phenomenon known as sperm heteromorphism (see [1,2] for detailed reviews). Each ejaculate contains both fertilizing eupyrene sperm and non-fertilizing apyrene sperm. Eupyrene spermatogenesis initiates first, during larval life. In early pupal development, a hormonally driven switch initiates apyrene spermatogenesis [3].

Eupyrene and apyrene sperm are morphologically very distinct. Eupyrene sperm are longer, they remain bundled together in the ejaculate, only becoming separate within the female genital tract [4]. The shorter, apyrene, sperm lack both a nucleus and an acrosome, explaining their inability to fertilize eggs. Both sperm morphs are motile, and this motility is required for normal reproduction [5,6]. Consistent with these dramatic differences in the final sperm, the process of spermatogenesis differs between the two morphs. Early primary spermatocytes are bi-potential; those in larval testes proceed along the eupyrene differentiation programme. Early pupae produce an as yet unidentified hormone, termed ‘apyrene spermatogenesis inducing factor’ (ASIF). On receipt of ASIF, spermatocytes that are still bi-potential switch programme and eventually differentiate into apyrene sperm. Already committed eupyrene spermatocytes do not respond to ASIF and remain on their eupyrene developmental trajectory [1]. Despite their different potential, primary spermatocytes on the two pathways are not morphologically distinguishable until they initiate the meiotic divisions.

Production of eupyrene spermatocytes involves a conventional meiosis I spindle, with robust microtubule arrays, a well-formed metaphase plate and segregation of homologous chromosomes in anaphase I [7]. In contrast, the meiosis I spindle in cells destined to become apyrene sperm is much less robust, with reduced microtubule arrays and a poorly defined metaphase plate. There is extensive chromosome non-disjunction leading to aneuploid cells with dispersed micronuclei. As eupyrene spermatids elongate, the nuclei cluster at one end of the cyst becomes needle-shaped and intimately associates with the overlying cyst cell. In contrast, micronuclei in apyrene spermatids cluster in the middle of the elongating cyst and are gradually degraded [8]. Spermatid individualization of both morphs involves peristaltic squeezing of cyst cells; removing excess cytoplasm from all spermatids and forcing elimination of nuclei from apyrene spermatids.

The final product of this deliberate and orchestrated process is two morphs with different morphologies made with different proteomes, comprising some shared proteins and some proteins unique to one or other morph [9,10]. A small number of genes have been demonstrated via CRISPR-Cas9/RNAi experiments to be important for lepidopteran sperm heteromorphism, including *Sex-lethal (Sxl*) (electronic supplementary material, table S1) [5,6,11,12]. However, there has not been a systematic, unbiased identification of differentially expressed genes that ensure normal differentiation of spermatocytes towards eupyrene and apyrene sperm fates.

To identify genes that may be required for the alternative differentiation trajectories, we compared the transcriptomes of spermatocytes from larval *Galleria mellonella* testes, that were destined to become eupyrene sperm, with spermatocytes from pupal testes that were destined to become apyrene sperm. A high number of differentially expressed genes were found, including transcription factors, meiotic regulators and sperm axoneme components. A comparison of our transcriptomic dataset with mature sperm proteomic data from other Lepidoptera [10], and subsequent phylogenetic analysis, enabled validation of our RNA-seq data, as consistent with the resulting mature sperm proteomes. Furthermore, phylogenetic analysis elucidated the evolution of eupyrene- and apyrene-enriched paralogues of both the sperm axoneme component *Ccdc63* and *β-tubulin* in moths, providing an insight into the evolution of lepidopteran sperm heteromorphism.

## 2. Material and methods

### 2.1. *Galleria mellonella* culture

Wild-type *G. mellonella* larvae were provided by the *Galleria mellonella* Research Centre (Exeter University) and initially stored in the dark at room temperature and maintained on a food medium based on diet 3 of [13] (electronic supplementary material, Methods section). Larvae were subsequently incubated at 30°C to induce pupation [13].

### 2.2. Testis spills

Individual follicles were dissected from three last instar larval testes and three pupal testes, cut open in 10 µl PBT (1 × PBS, 0.1% Tween 20) and pipetted onto a slide. Paraformaldehyde (10 µl of 4% w/v in PBT) was added for 10 min at room temperature, and then 1 µg/ml Hoescht (33 258) in mounting medium (2.5% *n*-propyl gallate in 85% glycerol,) was added. Fluorescence was analysed using Olympus Bx50 and images were taken with a Hamamatsu ORCA-05G camera and HCImage software.

### 2.3. Fluorescence hybridization chain reaction (HCR-FISH)

HCR-FISH v. 3 [14] was used to visualize mRNA expression in *G. mellonella* larval (*n* = 10) and pupal (*n* = 10) testes [14] (see electronic supplementary material, Methods 2 for detailed protocol) using 2–4 oligonucleotide probe pairs per gene (electronic supplementary material, table S2). Fluorescence was visualized using either the Zeiss Lightsheet Z.1 system or the Zeiss LSM880 Airyscan upright confocal microscope in the Cardiff Bioimaging Hub (electronic supplementary material, Methods section).

### 2.4. RNA-seq of *G. mellonella* primary spermatocyte cysts

Two primary spermatocyte cysts were collected from each of five sixth instar larvae and five 3-day-old pupae (electronic supplementary material, Methods section). RNA libraries were produced from the 20 primary spermatocyte cysts using the QIAseq FX Single Cell RNA Library Kit (Qiagen). Library quality and fragment size were assessed with the D1000 Tapestation (Agilent) and DNA size selection with the Blue Pippin system (Sage Science), by the Cardiff Genomics Research Hub. Libraries were sequenced on an Illumina NextSeq500 Sequencer (electronic supplementary material, Methods section).

### 2.5. Bioinformatics and statistical analysis

A standard RNA-seq bioinformatics pipeline comprising FastQC, Trimmomatic and Hisat2 was used to assess sequence quality, to align reads to the *G. mellonella* reference genome (CSIRO_AGI_GalMel_v. 1, Rahul Vivek Rane 2022) from NCBI [15]. Samtools and FeatureCounts were used to count reads that mapped to annotated genomic features (electronic supplementary material, Methods 5, table S3). Statistical analysis was carried out in R studio. SARTools R package [16] with DEseq2 (v. 1.38.3) was used for normalization of the data and differential gene analysis. Samples with low reads and/or ambiguous larval versus pupal clustering after principal component analysis (PCA) and hierarchal clustering, were removed before the final DEseq2 analysis. Heatmaps were created using normalized counts of top 100 upregulated differential gene expressions (DEGs) and top 100 downregulated DEGs using the ComplexHeatmap (v. 2.14.0) package with Pearson clustering [17]. All significant (*p *< 0.05; DEseq2 adjusts for multiple comparisons) DEGs were input into the DAVID Bioinformatics database gene conversion tool to obtain gene names [18]. DAVID functional annotation analysis was completed to obtain enriched gene ontology (GO) and keyword (KW) terms (*p *< 0.2).

### 2.6. Phylogenetic analysis of DEGs of interest

*Galleria mellonella* genes were initially screened for a potential follow-up analysis based on their absolute expression level, the fold change in gene expression between lineages and gene function predictions consistent with a role in spermatogenesis. The protein sequence of interest was input into BLASTP as a query sequence against *Galleria mellonella*, *Bombyx mori, Manduca sexta*, *Danaus plexippus, Drosophila melanogaster*, *Aedes aegypti* and *Homo sapiens* proteomes. Phylogenetic analysis was then completed using MEGA v. 11.0. 13 software [19] (electronic supplementary material, Methods section). The resulting phylogenetic trees were then cross-referenced with our RNA-seq dataset and previously published proteomic and transcriptomic datasets [10,20–23].

## 3. Results

### 3.1. Validation of the switch to apyrene sperm development in *G. mellonella* pupae

**Figure 1.**
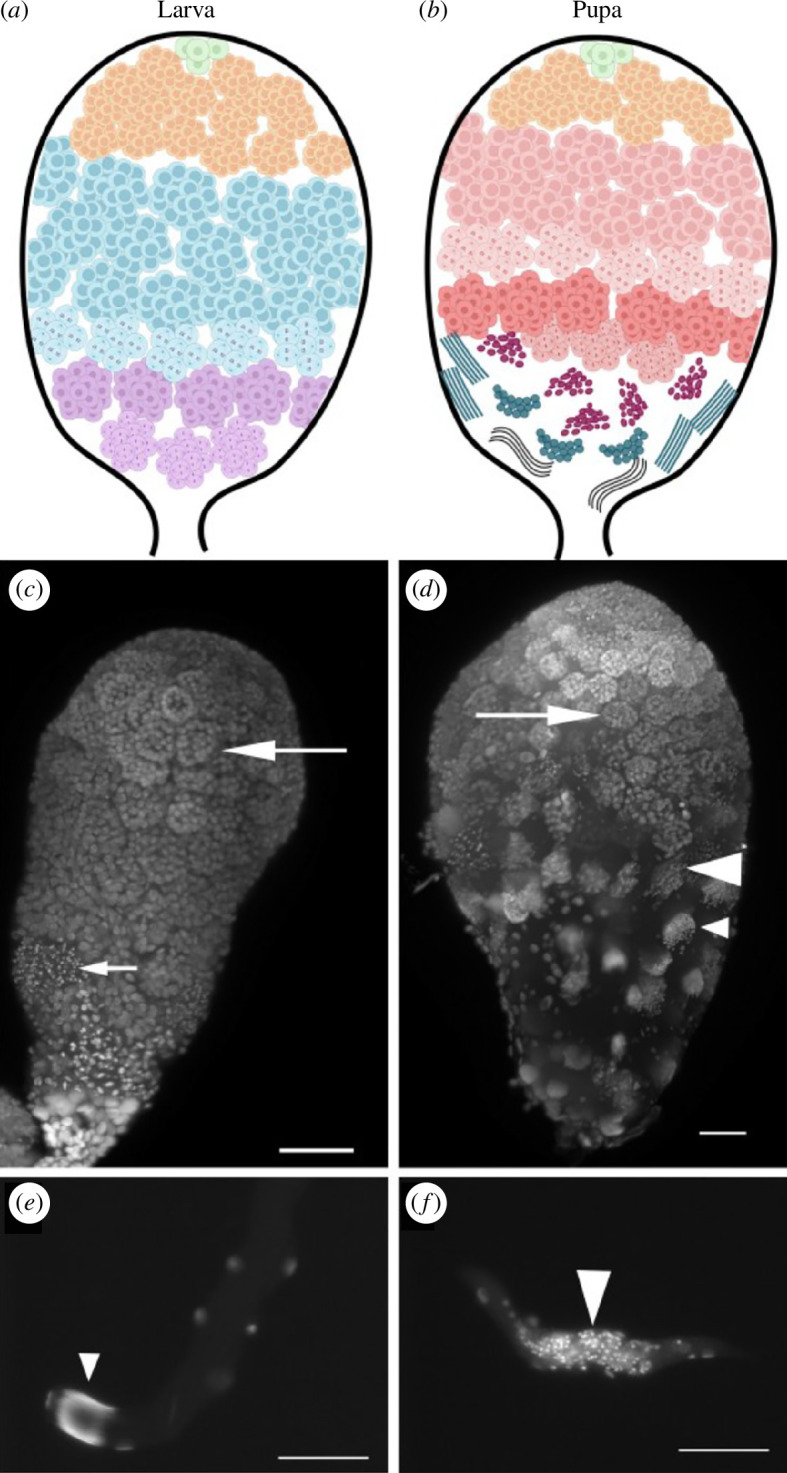
*G. mellonella* larval and pupal testis morphology. Schematic representations, and whole mount testes stained for DNA, of larval (*a, c*) and pupal (*b, d*) testes from *G. mellonella*. Germline stem cells and spermatogonia reside at the apical tip (green), and generate cysts of bi-potential early spermatocytes (orange) encapsulated by cyst cells. In larvae, these differentiate into late primary spermatocytes on the eupyrene sperm trajectory (blue in *a*, large arrow in *c*), which undergo meiosis and become secondary spermatocytes and then early spermatids (purple in *a*, small arrow in *c*). In pupal testes, late spermatocytes (pink in *b*, large arrow in *d*) differentiate along the apyrene sperm trajectory, to generate secondary spermatocytes and spermatids (dark pink in *b*, large arrowhead in *d*). Pupal testes also contain a few eupyrene secondary spermatocytes (small arrow in *d*) and spermatids (teal in *b*, small arrowhead in *d*). DNA staining of eupyrene spermatid (*e*) and apyrene spermatid (*f*) cysts, with nuclei at the end of the cyst (small arrowhead) or centrally located (large arrowhead) respectively. The scale bar is 50 μm.

To validate the switch to apyrene sperm production after the onset of pupation, individual testis follicles from *Galleria mellonella* sixth instar larvae and 3-day-old pupae were stained for DNA. Whole testes and spilled testis contents confirmed eupyrene spermatogenesis in larval stages (figure 1*a,c* and *e*) and apyrene spermatogenesis in pupal stages (figure 1*b,d* and *f*). Primary spermatocytes were morphologically indistinguishable between larval and pupal testes (figure 1*c,d*, large arrows). Early haploid eupyrene spermatids were observed in larval testis (figure 1*c*, small arrow), with spermatids subsequently completing spermiogenesis in pupal stages to form bundles with elongated nuclei (figure 1*d,e* small arrowheads). Importantly, early apyrene spermatids with centrally located nuclei were only observed in pupal testes (figure 1*d,e*, large arrowheads). Therefore, we concluded that primary spermatocytes collected from larvae and pupae were representative of eupyrene and apyrene development, respectively.

### 3.2. *Gmsxl* expression persists longer in cells progressing through the apyrene differentiation pathway

The RNA-binding protein Sxl is required for apyrene sperm development in *Bombyx mori,* but for the development of both sperm morphs in the tobacco cutworm, *Spodoptera litura* [5,6,24]. In *B. mori*, *Bmaly*, a homologue of the *D. melanogaster* meiotic transcriptional regulator *aly* and its paralogue *lin9*, is required for the progression of spermatocytes into the meiotic divisions in larval testes; its role in pupal testes has not been evaluated [25]. To evaluate the expression of these genes in both eupyrene and apyrene differentiation in *G. mellonella*, we used HCR-FISH on larval and pupal testes.

**Figure 2.**
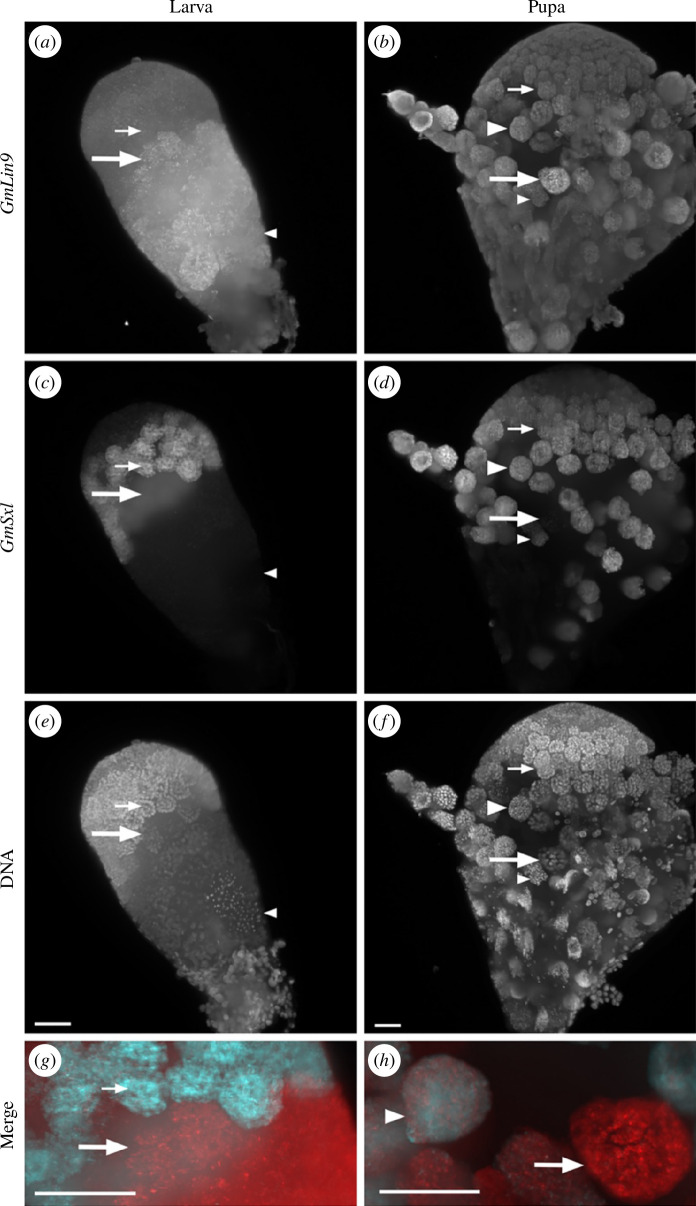
*Gmsxl* expression persists longer in the apyrene spermatogenesis programme. HCR-FISH analysis of Gmlin9 (*a*, *b*), red in merged images, (*g, h*) and *Gmsxl* (*c*, *d*), cyan in merged images (*g*, *h*), and DNA staining (*e, f*) of larval (*a, c, e, g*) and pupal (*b, d, f, h*) testes. *Gmlin9* expression is low and *Gmsxl* expression is high in early spermatocytes (small arrows). Spermatocyte cysts that will differentiate into eupyrene sperm have high *Gmlin9* and low *Gmsxl* (large arrow). Spermatocyte cysts that will differentiate into apyrene sperm have high *Gmlin9* and high *Gmsxl* (large arrowhead). Imaged using the Zeiss Lightsheet Z.1 system. The scale bar is 50 μm.

*Gmlin9* was expressed through both developmental trajectories. An abrupt increase in expression was found as spermatocytes matured in larval testes (figure 2*a*). In pupal testes, *Gmlin9* expression increased steadily, peaking in mature primary spermatocytes (figure 2*b*). *Gmlin9* transcript gradually declined through secondary spermatocytes and spermatids (figure 2*a,b*, small arrowheads). In larval testes, *Gmsxl* expression was high in late spermatogonia and early primary spermatocytes (figure 2*c,g*, small arrows), and the transcript abruptly declined in late spermatocytes (figure 2*c,g*, large arrows). No signal was detected in eupyrene cysts undergoing meiotic divisions (figure 2*c*, small arrowhead). Thus, cysts on the eupyrene developmental trajectory initially had high *Gmsxl* and low *Gmlin9* before switching to a low *Gmsxl*, high *Gmlin9*, state as late spermatocytes.

In pupal testes, *Gmsxl* was expressed in late spermatogonia and early spermatocytes (figure 2*d*, small arrow) and was detected at high levels in many late primary spermatocyte cysts (figure 2*d,h*, large arrowhead). A few very late spermatocytes lacked *Gmsxl* transcript (figure 2*d,h*, large arrow); these are probably cysts that had already committed to eupyrene differentiation before the early pupal action of ASIF. Secondary spermatocytes and spermatids destined to become apyrene sperm also retained some *Gmsxl* transcript (figure 2*d*, small arrowhead). Thus, cysts on the apyrene developmental trajectory initially had high *Gmsxl* and low *Gmlin9*, then had high *Gmsxl* and high *Gmlin9* before switching to a low *Gmsxl*, medium *Gmlin9*, state as early spermatids.

### 3.3. RNA-seq of primary spermatocytes from larval versus pupal testes

The differential expression of *Gmsxl* in spermatocytes on the two differentiation pathways confirms that these morphologically identical cells have different transcriptome profiles. We used an unbiased approach to investigate transcriptomic differences between primary spermatocyte cysts destined to become eupyrene sperm versus apyrene sperm. RNA-seq was conducted on individual primary spermatocyte cysts collected from larval (10 cysts) and pupal testes (9 cysts) (electronic supplementary material, figure S1). Results for trimming and subsequent mapping to the *G. mellonella* reference genome are shown in electronic supplementary material, table S3. Larval sample 5.2 was excluded at this stage owing to low mapping percentage (electronic supplementary material, table S3).

### 3.4. Larval and pupal cysts clustered separately in principal component analysis plots

**Figure 3.**
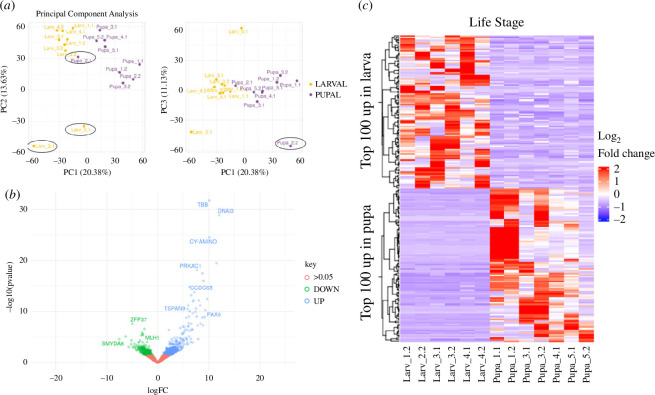
RNA-seq of larval and pupal spermatocytes reveals differential transcriptomes. (*a*) Principal component analysis of cyst sequencing. Cysts excluded from further analysis are circled. (*b*) A volcano plot of all detected genes comparing expression in larval cysts with pupal cysts reveals more genes significantly upregulated in pupal cysts. (*c*) Heat map of genes with highest fold changes, revealing the variability in signals in the different cysts comprising the whole sample.

The morphological examination and *Gmsxl* FISH both indicated that day 3 pupal testes contain a few late spermatocyte cysts that are on the eupyrene sperm differentiation pathway, having been early primary spermatocytes just past the commitment point when the early pupal ASIF induced switch occurred. To ensure unambiguous eupyrene and apyrene samples for valid DEG analysis, hierarchal clustering and PCA were conducted. Six larval samples and seven pupal samples were included in the final transcriptomic analysis. Two larval samples (2.1 and 5.1) clearly clustered away from other larval samples (figure 3*a*), while another larval sample (1.1) clustered within pupal samples in hierarchal clustering (electronic supplementary material, figure S2A). Pupal sample 2.1 clustered very closely to larval samples (figure 3*a*), and was potentially a eupyrene-destined primary spermatocyte. Pupal sample 2.2 also clustered away from other pupal samples when comparing PC_1_ and PC_3_ axes (electronic supplementary material, figure S2A). Therefore, these samples were removed from the final DEG analysis to ensure a biologically valid comparison (electronic supplementary material, figure S2B). DEG analysis using DESeq2, with an adjusted *p*-value threshold of <0.05, identified 373 genes significantly upregulated in primary spermatocytes from larvae, and 686 genes significantly upregulated in pupal primary spermatocytes (figure 3*b*; electronic supplementary material, data files 1, 2 and 3). While we did not impose a fold change cut-off, in practice all bar one of these differentially expressed genes showed a twofold or more difference between cyst types.

### 3.5. DAVID functional enrichment analysis highlighted genes involved in core biological processes in spermatogenesis

The DEG lists were input into DAVID functional enrichment analysis (see electronic supplementary material, data file 4 for full lists). Terms such as ‘transcription’, ‘DNA binding’ and ‘zinc ion binding’ were enriched in larval spermatocytes, while ‘cell division’, ‘motile cilium’ and ‘coiled-coil’ were enriched in pupal spermatocytes (electronic supplementary material, data file 4). These terms are biological processes and protein properties expected to appear in sperm development, and there is no obvious enriched term to explain the fertile versus infertile sperm fate decision in early spermatocytes. The genes enriched in the GO and KW terms were then investigated further via literature analysis. Several were previously known to be involved in spermatogenesis, summarized in electronic supplementary material, table S4. Overall, our DEG analysis has revealed many genes of interest involved in core biological processes in spermatogenesis that are differentially expressed between the early eupyrene- and apyrene-committed cells, with a large scope for future exploration.

We examined the RNA-seq data for *Gmlin9* and *Gmsxl* genes, to evaluate if the HCR-FISH and RNA-seq data were consistent. As expected from HCR-FISH analysis, *Gmlin9* (LOC113523285) was not differentially expressed between larval and pupal spermatocytes (figure 2; electronic supplementary material, tables S4 and S5). Interestingly, *Gmsxl* (LOC113515001) was upregulated in pupal spermatocytes, supporting the HCR-FISH finding that *Gmsxl* expression persists to a later stage in the apyrene sperm development (figure 2; electronic supplementary material, table S5). However, this upregulation was not significant (Log2FC = 1.475, *p*.adj = 0.081399). Overall, the corroboration of *Gmsxl* and *Gmlin9* HCR-FISH expression patterns in *G. mellonella* testes and RNA-seq expression values validates the predictive value of our RNA-seq dataset.

We also investigated RNA-seq results for other previously discovered sperm heteromorphism regulators in Lepidoptera (electronic supplementary material, table S1). None were differentially expressed between larval and pupal spermatocytes. Interestingly, two genes implicated in sperm heteromorphism regulation in *B. mori* (*Maelstrom* and *PNLDC1*) were barely detected in sequenced *G. mellonella* primary spermatocyte cysts, suggesting differences between lepidopteran species (electronic supplementary material, table S1).

### 3.6. *GmTaf4* is expressed at higher levels in larval than pupal primary spermatocytes

**Figure 4.**
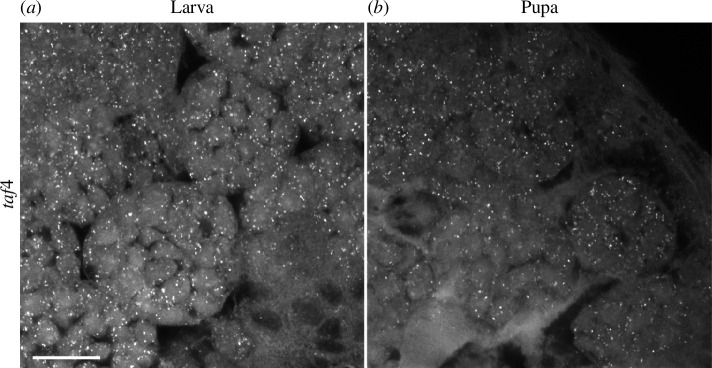
*Taf4* expression is higher in larval spermatocytes than pupal spermatocytes. HCR-FISH analysis of *GmTaf4* in larval (*a*) and pupal (*b*) spermatocytes. Imaged using the same acquisition settings for both samples on the Zeiss LSM880 Airyscan upright confocal microscope. The scale bar is 20 μm.

Among the DEGs contributing to the ‘transcription’ annotation term enrichment in larval spermatocytes was LOC113519479, which encodes Taf4, a subunit of the general transcription factor complex TFIID. In *D. melanogaster* a testis-specific paralogue of *Taf4*, *nht*, is critical for testis-specific transcription [26]. BLAST searches confirmed that this is a single copy gene in *G. mellonella* and other sequenced Lepidoptera. A difference in expression of this gene could result in higher total transcriptional activity in larval spermatocytes compared to pupal spermatocytes. HCR-FISH showed that *taf4* is expressed in primary spermatocytes in both larval and pupal testes, as expected given its critical role in transcription, but also confirmed that the transcript was more abundant in late larval spermatocytes than pupal spermatocytes at the same differentiation stage (figure 4).

### 3.7. Gene duplication and specialization have produced apyrene- and eupyrene-enriched *Ccdc63* paralogues in moths

**Figure 5.**
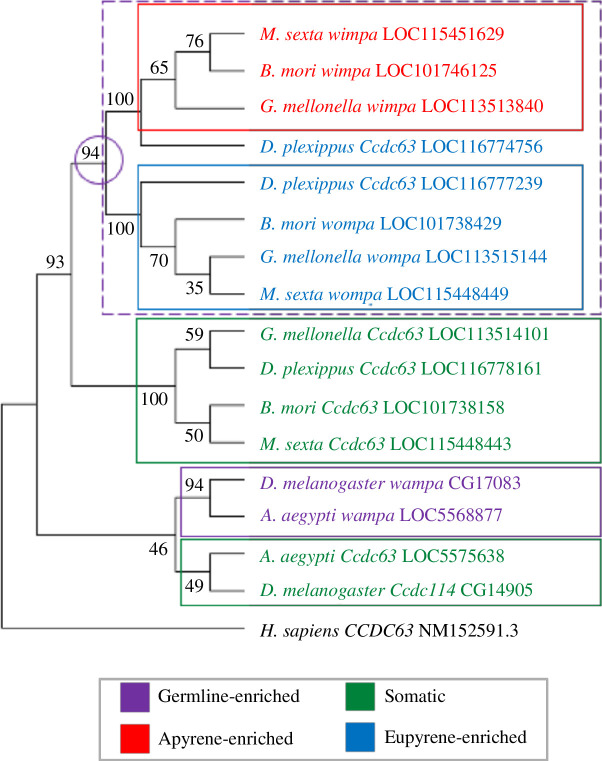
*Ccdc63* phylogenetic tree reveals gene duplication and sub-functionalization events in Lepidoptera. The maximum likelihood tree of conserved Ccdc63 protein regions. 16 Ccdc63 homologues were analysed from lepidopteran (*G. mellonella, D. plexippus, M. sexta, B. mori*) and dipteran (*D. melanogaster, A. aegypti*) species, with human (*H. sapiens*) CCDC63 as the outgroup. Both lepidopteran and dipteran species have evolved germline (purple box) and somatic (green box) paralogues. However, the lepidopteran germline gene underwent a further gene duplication to produce sperm-morph-specific paralogues. For moth species, paralogues evolved apyrene-specific (red box) and eupyrene-specific (blue) functions. The monarch butterfly *D. plexippus* appears to have evolved two eupyrene-specific paralogues (blue text). Bootstrap values (100 repeats) are shown.

One of the most dramatically upregulated genes in pupal spermatocytes was coiled-coil domain containing protein 63 (*Ccdc63,* LOC113513840) (figure 3*b*; electronic supplementary material, table S4). Ccdc63 is a component of the outer dynein arm docking complex, involved in the formation of the sperm axoneme. Phylogenetic analysis of *Ccdc63* evolution, incorporating lepidopteran and dipteran species, revealed a series of gene duplication and sub-functionalization events, to produce somatic-, germline- and morph-enriched paralogues. In Diptera, a duplication of the ancestral gene generated a somatically expressed paralogue and a germline-expressed paralogue. In *D. melanogaster*, these are *Ccdc114* (*CG14905*), expressed in Johnston’s organ neurons which possess motile cilia, and *wampa* [23,27], which encodes a component of the sperm proteome [28], respectively. In *A. aegypti,* AAEL011965 (LOC5575638) is highly expressed in the antenna [29] while the *wampa* orthologue, AAEL007188 (LOC5568877) is highly expressed in the testis [20,21]. In Lepidoptera, a similar but independent duplication of the ancestral gene generated somatic- and germline-enriched paralogues (figure 5). Interestingly, the post-duplication germline gene underwent a further duplication to give two germline-enriched paralogues in all lepidopteran species analysed. *M. sexta* sperm proteomic data revealed specialization of one germline paralogue for eupyrene sperm and the other paralogue for apyrene sperm [10]. The *M. sexta* apyrene-enriched protein (LOC115451629) clustered in the phylogenetic tree with *G. mellonella Ccdc63* (LOC113513840), which was highly expressed in apyrene-destined spermatocytes (red cluster, figure 5). Further evidence to support the specialization of these paralogues in moths is provided by transcriptomic data from *B. mori* larvae that detected enrichment of predicted eupyrene-enriched *Ccdc63* (LOC101738429) transcripts in larval testes, but not *Ccdc63* (LOC101746125) transcripts predicted to be apyrene-enriched (higher expression in pupal testes) [22]. This apyrene specialization may be exclusive to moths, as the paralogous protein in the monarch butterfly *D. plexippus* (LOC116774756) was found to be enriched in eupyrene sperm, rather than apyrene [10]. Based on the name of the *D. melanogaster* homologue, *wampa* [27], we named the largely apyrene-enriched paralogue (LOC113513840) *wimpa*, and the eupyrene-enriched paralogue (LOC113515144) *wompa*.

**Figure 6.**
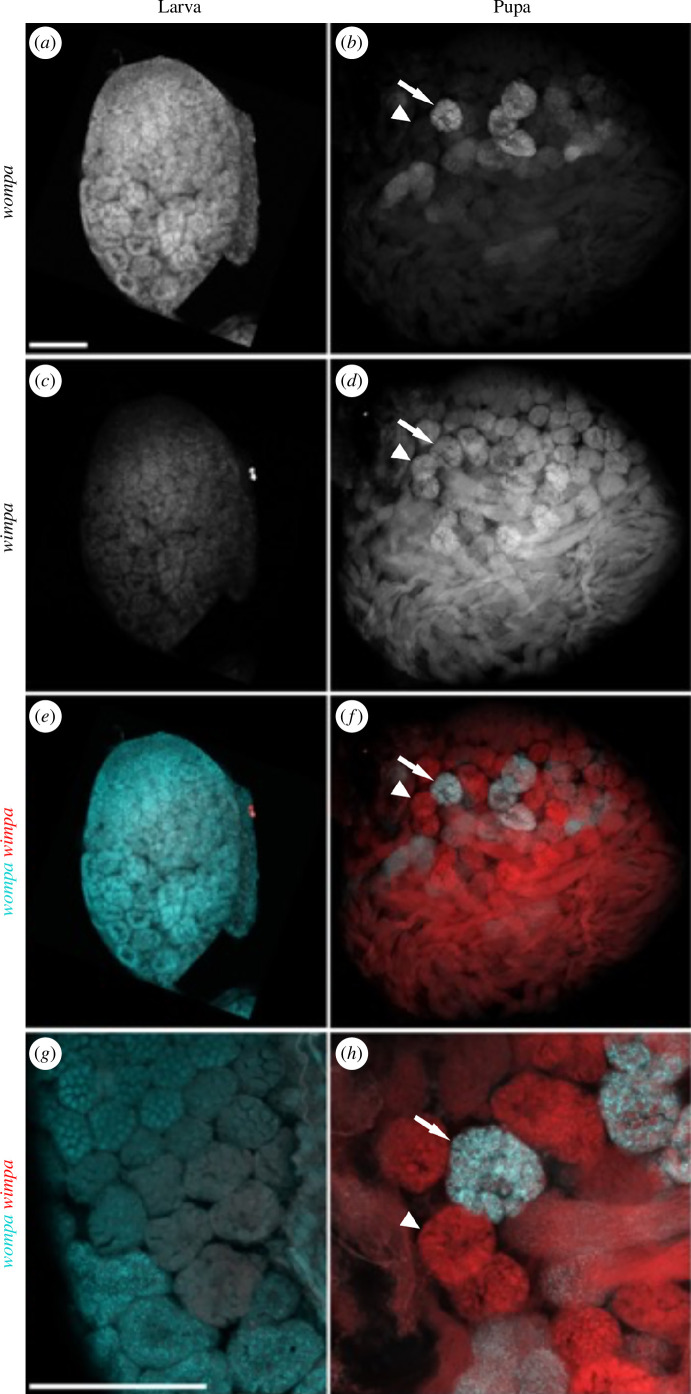
Differential expression of *Ccdc63* paralogues *wimpa* and *wompa* between developing apyrene and eupyrene sperm in *G. mellonella*. HCR-FISH analysis of *wompa* LOC113515144 (*a*, *b)*; cyan in merged images *(e–h*) and apyrene-enriched *wimpa* LOC113513840 (*c*, *d*); red in merged images (*e–h*) of larval (*a, c, e, g*) and pupal (*b, d, f, h*) testes. High *wompa* and low *wimpa* expression were detected in larval eupyrene-destined spermatocytes (*a, c*). *Wimpa* was highly expressed in spermatocytes and spermatids on the apyrene differentiation pathway in pupal testes (*d*). Predicted eupyrene-committed cysts in pupal testes demonstrated co-expression of both *wompa* and *wimpa* (large arrow), while predicted apyrene-committed cysts had *wimpa* expression only (arrowhead). Imaged using Zeiss LSM880 Airyscan upright confocal microscope. The scale bar is 100 μm.

HCR-FISH revealed the expression patterns of *wompa* (LOC113515144; eupyrene) and *wimpa* (LOC113513840; apyrene) in *G. mellonella* larval and pupal testes. This confirmed a general pattern of high expression of *wompa* in larval spermatocytes, and high expression of *wimpa* in pupal spermatocytes, as expected from phylogenetic analysis (figure 6). The clear upregulation of *wimpa* in pupal testes versus larval testes also corroborated the RNA-seq data (electronic supplementary material, tables S45). HCR-FISH revealed *wimpa* and *wompa* co-expression in a small number of pupal cysts, predicted to be spermatocyte cysts committed to the eupyrene pathway (figure 6). *Wompa* was relatively highly expressed across all primary spermatocyte samples in our RNA-seq dataset, with higher variation between pupal spermatocyte cyst samples (electronic supplementary material, table S5). This could indicate that both *wompa* and *wimpa* paralogues are important in the later stages of eupyrene sperm development, while *wimpa* alone is necessary for apyrene sperm development.

### 3.8. Divergent expression of *β-tubulin* family members in Lepidoptera

From our RNA-seq data, another gene significantly upregulated in pupal spermatocytes was a β-tubulin gene (LOC113522729) of unknown genealogy (electronic supplementary material, table S4). Many β-tubulin genes (e.g. LOC113519435) were also among the most highly expressed in the spermatocyte transcriptomic data (electronic supplementary material, data file 3). β-tubulin proteins, along with α-tubulin, constitute the microtubule cytoskeleton, which acts as a structural framework in cells, crucial for cell morphology, cell division, intracellular transport and axoneme formation. In *D. melanogaster*, there are five β-tubulin genes. β*2* (*β-tubulin 85D*) exclusively expressed in the male germline, is required for successful meiosis and axoneme elongation in spermatogenesis and is abundant in the sperm proteome [28,30,31]. *β-tubulin 65B* is also expressed exclusively in the male germline, but its role in spermatogenesis has not been determined, and the protein has not been detected in the sperm proteome [28]. β1 (*β-tubulin 56D*) is expressed in both soma and male germline, and detected in the sperm proteome, while expression of both β3 (*β-tubulin 60D*) and β4 (*β-tubulin 97EF*) is restricted to the soma. *Aedes aegypti* has a similar β-tubulin gene tree but has evolved two β4-tubulin paralogues [32]. In *Bombyx mori*, four β-tubulin family members have described, including two somatic β1-tubulin paralogues (β1a and β1b), testis-specific β2-tubulin and somatic β3-tubulin. Whittington *et al.* [10] [10] identified six β-tubulin proteins in the mature sperm proteomes of both *M. sexta* and *D. plexippus,* with differing sperm morph specificity. In *M. sexta,* one β-tubulin protein was apyrene-enriched while the remaining β-tubulin proteins were detected in both sperm morphs. Contrastingly, in *D. plexippus,* two eupyrene-specific β-tubulin proteins were identified, alongside one apyrene-specific β-tubulin protein and three shared proteins. We used phylogenetic analysis to resolve the evolutionary relationships of the sperm-morph-specific β-tubulin proteins detected by Whittington *et al.* [10], and the apyrene-specific β-tubulin *G. mellonella* gene.

**Figure 7.**
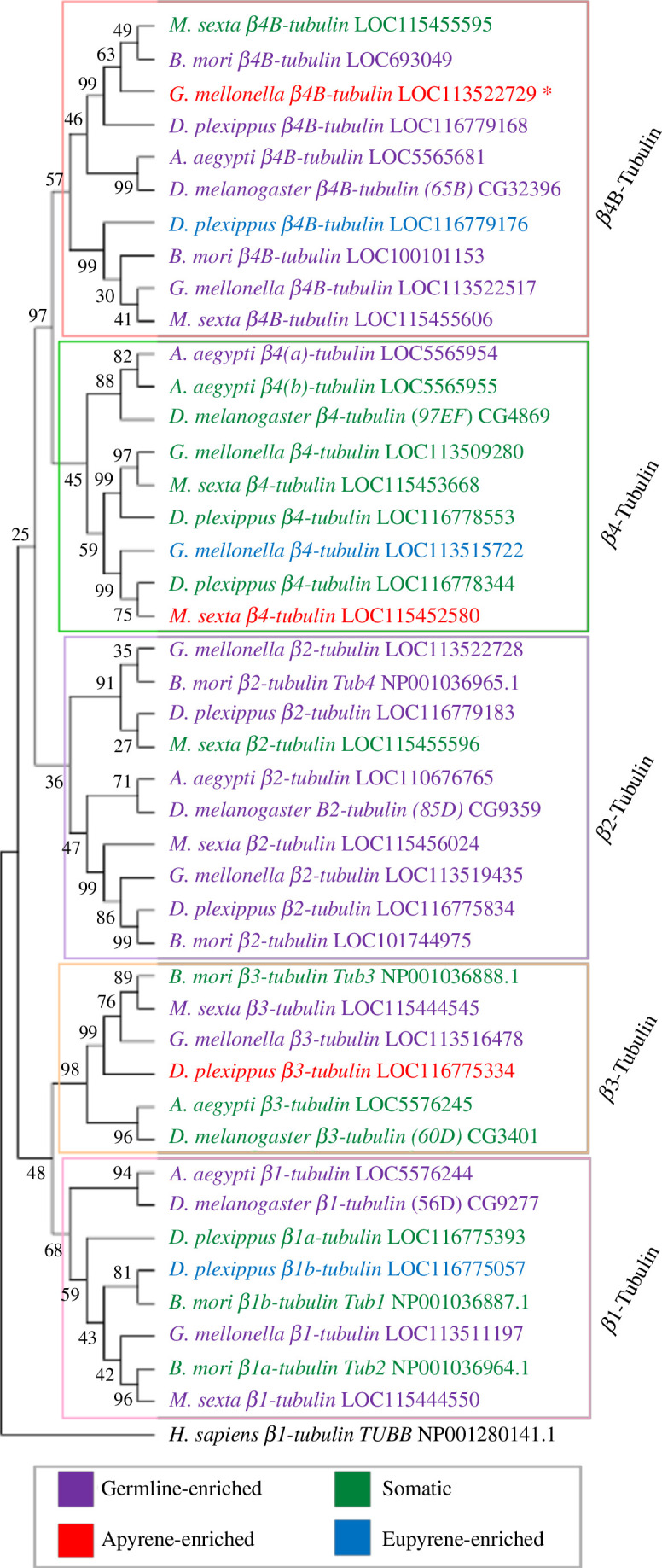
Phylogenetic analysis of the lepidopteran β-tubulin family. The neighbour-joining tree of 42 β-tubulin proteins from Lepidoptera (*G. mellonella, D. plexippus, M. sexta, B. mori*) and Diptera (*D. melanogaster, A. aegypti*), with human (*H. sapiens*) β1-tubulin as the outgroup. Boxes outline the different β-tubulin family members. The β-tubulin gene significantly upregulated (*p *< 0.05) in *G. mellonella* pupal spermatocytes is indicated by the red asterisk. Text colours denote the expression pattern based on available transcriptomic and proteomic data: apyrene-enriched (red), eupyrene-enriched (blue), germline-enriched (purple) or somatic (green). Bootstrap values (10 000 repeats) are shown.

A neighbour-joining phylogenetic tree of β-tubulin protein sequences is shown in figure 7. The identification of the major subfamilies was via published assignments and confirmed by analysis of the C-terminal sequences (electronic supplementary material, figure S3). Before the divergence of Lepidoptera and Diptera, a duplication of the ancestral β*4-tubulin* gene (green circle, figure 7), produced β*4-tubulin* (green box, figure 7) and β*4B-tubulin* (red box, figure 7). Subsequent lepidopteran-specific gene duplications expanded the β4*-tubulin* family. The apyrene-specific β-*tubulin* gene (LOC113522729) from our RNA-seq (red asterisk, figure 7) is a semi-orthologue of the poorly characterized, germline-specific *β-tubulin 65B* from *D. melanogaster* (CG32396); both are β*4B-tubulins*. While many β*4-tubulin* genes had low expression in the male germline, almost all the lepidopteran β*4B-tubulin* paralogues were highly expressed in the male germline [10,33] (red box, figure 7). The exception was *M. sexta* β*4B-tubulin* (LOC115455595), which was assumed to be somatic or lowly expressed owing to its absence from the sperm proteomic dataset. In contrast to *wimpa/wompa*, the β*4B-tubulin* paralogue lineages in Lepidoptera have not evolved clear apyrene- or eupyrene-enriched expression patterns, suggesting that the β*4B-tubulin* paralogues have independently acquired specific functions in sperm morph differentiation.

β*1-tubulin* and β*3-tubulin* family members in Lepidoptera also demonstrated a divergence in expression patterns between lepidopteran species (figure 7; electronic supplementary material, data file 3). In contrast, almost all β*2-tubulin 85D* orthologues were expressed in the germline, with *G. mellonella* β*2-tubulin 85D* genes (LOC113519435, LOC113522728) showing very high expression levels in all spermatocyte samples (purple cluster, figure 7; electronic supplementary material, data file 3). Phylogenetic analysis suggested that there are two β*2-tubulin 85D* paralogues in Lepidoptera, however, the bootstrap value is relatively low for this gene duplication (purple circle, figure 7).

**Figure 8.**
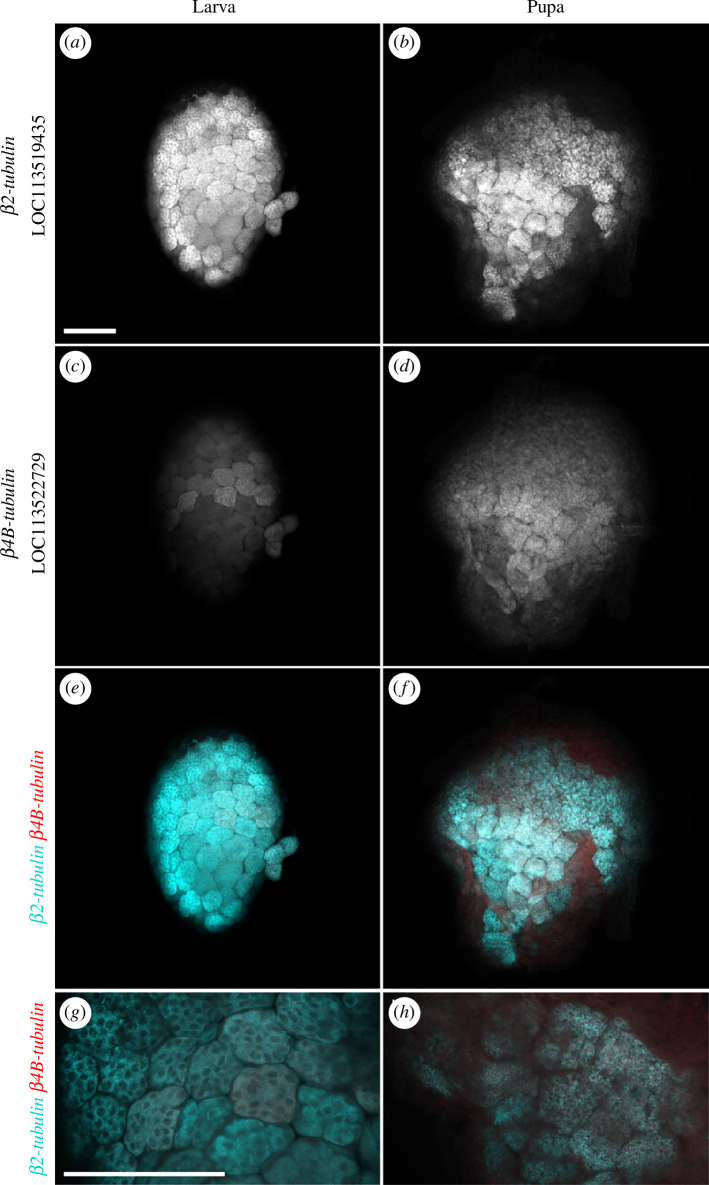
Ubiquitous versus apyrene-enriched expression of β*2-tubulin* and β*4B-tubulin* in *G. mellonella* testes. HCR-FISH analysis of β*2-tubulin* LOC113519435 (*a*, *b)*; cyan in merged images (*e–h*) and apyrene-enriched β*4B-tubulin* LOC113522729 (*c*, *d*); red in merged images (*e–h*), of larval (*a, c, e, g*) and pupal (*b, d, f, h*) testes. High levels of β*2-tubulin* were detected in both larval and pupal testes, corroborating ubiquitous germline expression (*a, b*). β*4B-tubulin* expression was higher in pupal testes versus larval (*c,d*), with low β*-tubulin* expression detected in a subset of eupyrene-destined primary spermatocytes in larval testes (*c*). Imaged using Zeiss LSM880 Airyscan upright confocal microscope. The scale bar is 100 μm.

HCR-FISH analysis confirmed the results from the RNA-seq analysis. The β*2-tubulin 85D* orthologue (LOC113519435) expression was detected at very high levels in spermatocytes and spermatids in both larval and pupal testes, suggesting that it is required for the differentiation of both sperm morphs (figure 8*a,b*; electronic supplementary material, table S5). In contrast, *in situ* staining of apyrene-specific β4B-tubulin (LOC113522729) in *G. mellonella* testes revealed increased expression in pupal spermatocytes and spermatids versus larval spermatocytes (figure 8*c,d*; electronic supplementary material, table S5). A low expression level of the predicted apyrene-specific β4B-tubulin (LOC113522729) was detected by HCR-FISH in a subset of eupyrene primary spermatocyte cysts in the larval testes, with RNA-seq analysis also detecting a very low level (figure 8*c*; electronic supplementary material, table S5). This suggests that β*4B-tubulin* (LOC113522729) is of particular importance in apyrene sperm differentiation in *G. mellonella*. Overall, phylogenetic and expression analysis of β*-tubulin* genes revealed lepidopteran-specific gene duplications that have not been previously identified. These β*-tubulin* duplications generated a suite of genes available for specialization for different sperm morphs, but there was a surprising variability of expression patterns of paralogues with respect to sperm morphs between different moth and butterfly species.

## 4. Discussion

Sperm heteromorphism is present in almost all Lepidoptera, and the production of two sperm morphs is essential for the fertility of moths and butterflies [1,5,6]. The non-fertilizing morphs are generated through precise, regulated processes, rather than through a variety of defective deviations from ‘normal’. While the proteomes of the sperm morphs contain many shared proteins, eupyrene and apyrene sperm also contain unique, specialized proteins [10]. How these differences are established earlier in spermatogenesis has not previously been described. In principle, the production of two similar, but distinct, final cell morphologies can depend on genes: (i) expressed in both lineages, with the same timing but different absolute levels; (ii) expressed exclusively in one or other lineage; (iii) expressed in both lineages but with different temporal dynamics; and (iv) duplicated and sub-functionalized, such that the required protein function is provided by distinct isoforms. We validated examples of (i) (*Taf4*), (iii) (*sxl*) and (iv) (*wompa/wimpa*, and *β-tubulin*). With our sequencing data, we cannot be sure of genes *exclusively* in one or other lineage, however, we did find examples of single copy genes with dramatic differences in absolute expression level between cyst types. LOC113516308, a gene conserved across arthropods, with no known or predicted function had >16-fold higher expression in larval spermatocytes than pupal spermatocytes. Meanwhile, LOC116412852, which encodes a predicted plasma-membrane associated CAP-domain containing protein conserved across Lepidoptera, had >1000-fold higher expression in pupal spermatocytes than in larval spermatocytes.

Our data confirm differential transcription of many genes, thus confirming that the unique sperm morphology is underpinned by differential transcription at the spermatocyte stage. Higher or lower expression of the general transcription factor Taf4, along with the differential expression of other transcriptional regulators, may be implicated in establishing the distinct transcriptomes. The persistence of *Sxl* expression in the apyrene spermatocytes could affect RNA stability or could result in the production of alternative splice variants in these cells. Among the DEGs, we found several that could regulate or enact the alternative meiosis seen in the apyrene differentiation programme (e.g. spindle proteins, cell cycle checkpoint proteins and meiotic recombination factors). Additionally, our DEG list includes many more examples of genes probably implicated in the differential elongation and final morphology. The validation of our RNA-seq dataset via phylogenetic analysis and identification of orthologous genes in published proteomic/transcriptomic datasets demonstrates the predictive nature of early transcriptomic differences on the final proteomes and thus the morphology of the mature sperm.

Phylogenetic analysis revealed the expansion of gene families in Lepidoptera via gene duplications, allowing for the paralogues to adopt specialized functions in producing the sperm heteromorphism phenotype. For example, the duplication of the ancestral sperm axoneme component *Ccdc63* [27] produced two paralogous genes with distinct expression patterns in *G. mellonella* larval and pupal testes. They are predicted to play unique roles in eupyrene and apyrene sperm development and have been termed *wompa* and *wimpa*, respectively. Ccdc63 has been duplicated to generate paralogues specifically for somatic axonemes and sperm axonemes in both Lepidoptera and Diptera [27], in addition to the sperm morph duplication in Lepidoptera. It may provide subtly different, but evolutionarily important, functionality to these distinct motile cilia, and comparative functional studies could be very interesting in the future. RNAi or CRISPR mutagenesis could be used to assess the roles of both *wompa* and *wimpa* in the two sperm lineages, as has been done for other genes in *B. mori* [12,25,34]. Comparative assays could include expression swap experiments, for example, expression of *wompa* under the control of the *wimpa* regulatory sequences, and the subsequent assessment of sperm motility and ultrastructure. A technically more straightforward cross species comparison may also be revealing, for example assessing the ability of *Galleria wompa* or *wimpa* to rescue the fertility defects caused by loss of function of *Drosophila wampa*.

A similar expansion of the well-known β*-tubulin* gene family was also observed in the lepidopteran species studied. Lepidopteran-specific gene duplications of ancestral β*4B-tubulin* and β*2-tubulin* genes have led to many germline-enriched β*-tubulin* genes. Again, we predict that these paralogues have undergone sub-functionalization to play key roles in sperm heteromorphism evolution, and in the normal function of the different morphs. Although there is evidence for sperm-morph enriched expression for a small number of β*-tubulin* genes, overall β*-tubulin* paralogues have not evolved a specific bias towards fertile or infertile sperm development, as found for *wimpa/wompa* paralogues. This suggests that the exact function of the β*4B-tubulin* and β*2B-tubulin* paralogues in enacting or regulating dichotomous spermatogenesis varies between lepidopteran species, revealing evolutionary flexibility in the co-option of genes in the process.

Interestingly, our RNA-seq analysis found a higher number of upregulated genes in pupal spermatocytes compared to larval, which contrasts with previous findings that apyrene sperm possess a *less* diverse proteome [10]. It also contrasts with the hypothesis that apyrene sperm present a functionally streamlined version of a eupyrene ancestor sperm present early in the lineage when dichotomous spermatogenesis evolved [2]. While some of the differences could be species-specific, we note that Whittington *et al.* [10] investigated mature sperm proteomes, whereas we have focused on the earlier spermatocyte transcriptome [10]. During dynamic processes, such as differentiation, there may be only a moderate correlation between transcript and protein levels owing to post-transcriptional regulation, providing a possible explanation for the observed disconnect [35]. Increased expression of regulatory proteins, that are required at higher levels in the apyrene spermatocytes during spermatogenesis, but are not included in the final mature sperm, could also contribute to the observed pattern. Furthermore, we hypothesize that the higher transcriptomic diversity of developing apyrene sperm may make evolutionary sense in an extension of the ‘out of the testis’ phenomenon [36]; apyrene spermatocytes may provide a playground to experiment with newly evolved genes in an environment with lower functional constraints in comparison with the fertilizing eupyrene sperm. Differential signatures of selection have already been described for proteins differentially expressed between morphs, with apyrene sperm-specific proteins showing little evidence of positive selection [37]. Our data on the differential expression of paralogous genes, and the variability of these between species, suggest ongoing selection acting on gene duplications, expression and sequence to ensure male fertility.

Our lepidopteran model of sperm heteromorphism, the wax moth *Galleria mellonella*, is an emerging model organism within the life sciences, predominantly as a model species for investigating the response to infection [38]. Genetic tools such as transgenesis and CRISPR-Cas9 genome editing are actively being developed in *Galleria mellonella*, making it an attractive model species for future research into sperm heteromorphism regulators [39].

Fundamental research into lepidopteran reproduction has potentially wide-reaching implications in terms of controlling lepidopteran pests in agriculture. Lepidopteran pests cause significant economic damage owing to crop loss, with the problem increasing owing to globalization causing further spread of invasive lepidopteran species [40]. Moreover, the wax moth *Galleria mellonella* is a significant pest of honeybees [41]. With the importance of biodiversity becoming increasingly prevalent in the public consciousness, innovative solutions are required to effectively target these pest populations. Seth *et al*. [2] recently proposed the presence of infertile apyrene sperm as a potential ‘Achilles heel’ to specifically target lepidopteran pests [2]. If one could engineer a strain that made normal apyrene sperm, but lacked eupyrene sperm, they should have good viability, mating competitiveness and induction of post-mating responses and yet be fully sterile. Our dataset provides a resource to mine and investigate novel genes implicated in sperm differential morphogenesis, and specifically target genes involved in either eupyrene or apyrene sperm development. Since our transcriptomic data were generated from spermatocytes, they can be used to identify genes actively transcribed at this stage, in either lineage, and thus provide a source of potential regulatory sequences to use for the design of homing gene drive, precision-guided sterile insect technique or ectopic expression systems, as well as potential targets for an RNAi based strategy. On the other hand, an improved understanding of lepidopteran reproduction could be important in the broader context of declines in lepidopteran pollinator populations [42]. The possible future decline in fertility (and hence survival) of moth and butterfly pollinator populations owing to these environmental factors may have devastating impacts on our ecosystems and global food production.

## Data Availability

The RNAseq datasets are available on NCBI SRA, accession number PRJNA1028403. Supplementary material is available online [43].
